# Supplementary material for: A toolkit for testing membrane-localising tags across species
Source: bioRxiv. 2025 Oct 22:2024.11.12.623055. Preprint. [Version 2] doi: 10.1101/2024.11.12.623055 (PMC12633465; doi:10.1101/2024.11.12.623055)
Supplement: Supplementary file 1 [file NIHPP2024.11.12.623055v2-supplement-1.pdf]

bioRxiv preprint doi: https://doi.org/10.1101/2024.11.12.623055; this version posted October 22, 2025. The copyright holder for this preprint (which was not certified by peer review) is the author/funder, who has granted bioRxiv a license to display the preprint in perpetuity. It is made available under aCC-BY 4.0 International license.

Supporting Information

for Karapidaki et al. (2025) A toolkit for testing membrane localising tags across species

Supplementary Figures

- Figure S1. Quantification of cell membrane localisation in the tunicate *Phallusia mammillata*
- Figure S2. Quantification of cell membrane localisation in the sea urchin *Paracentrotus lividus*
- Figure S3. Localisation of membrane-tagged reporters in the beetle *Tribolium castaneum*
- Figure S4. Quantification of cell membrane localisation in the crustacean *Parhyale hawaiensis*
- Figure S5. Localisation of membrane-tagged reporters in the nematode *Caenorhabditis elegans*
- Figure S6. Localisation of membrane-tagged reporters in annelid *Platynereis dumerilii* embryos
- Figure S7. Localisation of membrane-tagged proteins in annelid *Platynereis dumerilii* larvae
- Figure S8. Localisation of membrane-tagged reporters in the flatworm *Macrostomum lignano*
- Figure S9. Quantification of cell membrane localisation in hydrozoan *Clytia hemisphaerica* embryos
- Figure S10. Localisation of membrane-tagged reporters in hydrozoan *Clytia hemisphaerica* polyps
- Figure S11. Localisation of membrane-tagged reporters in scyphozoan *Pelagia noctiluca* embryos
- Figure S12. Localisation of membrane-tagged reporters in scyphozoan *Pelagia noctiluca* planulae
- Figure S13. Localisation of membrane-tagged reporters in the anthozoan *Nematostella vectensis*
- Figure S14. Localisation of tagged proteins in abnormal *Paracentrotus* embryos
- Figure S15. Improved reporters using endogenous UTRs and codon optimisation in *Clytia*

Supplementary Video

- Video S1. Dynamics of SP-CD8tm-mScarlet3 localisation (#10) in *Parhyale* embryos

bioRxiv preprint doi: https://doi.org/10.1101/2024.11.12.623055; this version posted October 22, 2025. The copyright holder for this preprint (which was not certified by peer review) is the author/funder, who has granted bioRxiv a license to display the preprint in perpetuity. It is made available under aCC-BY 4.0 International license.

Figure S1. Quantification of cell membrane localisation in the tunicate *Phallusia mammillata*

(Top) Same as in Figure 2, but images were captured and displayed with the same settings, to reveal differences in fluorescence intensity. Scale bar, 25 µm.

(Bottom) Quantification of fluorescence at the plasma membrane and in the cell interior, as described in the Methods.

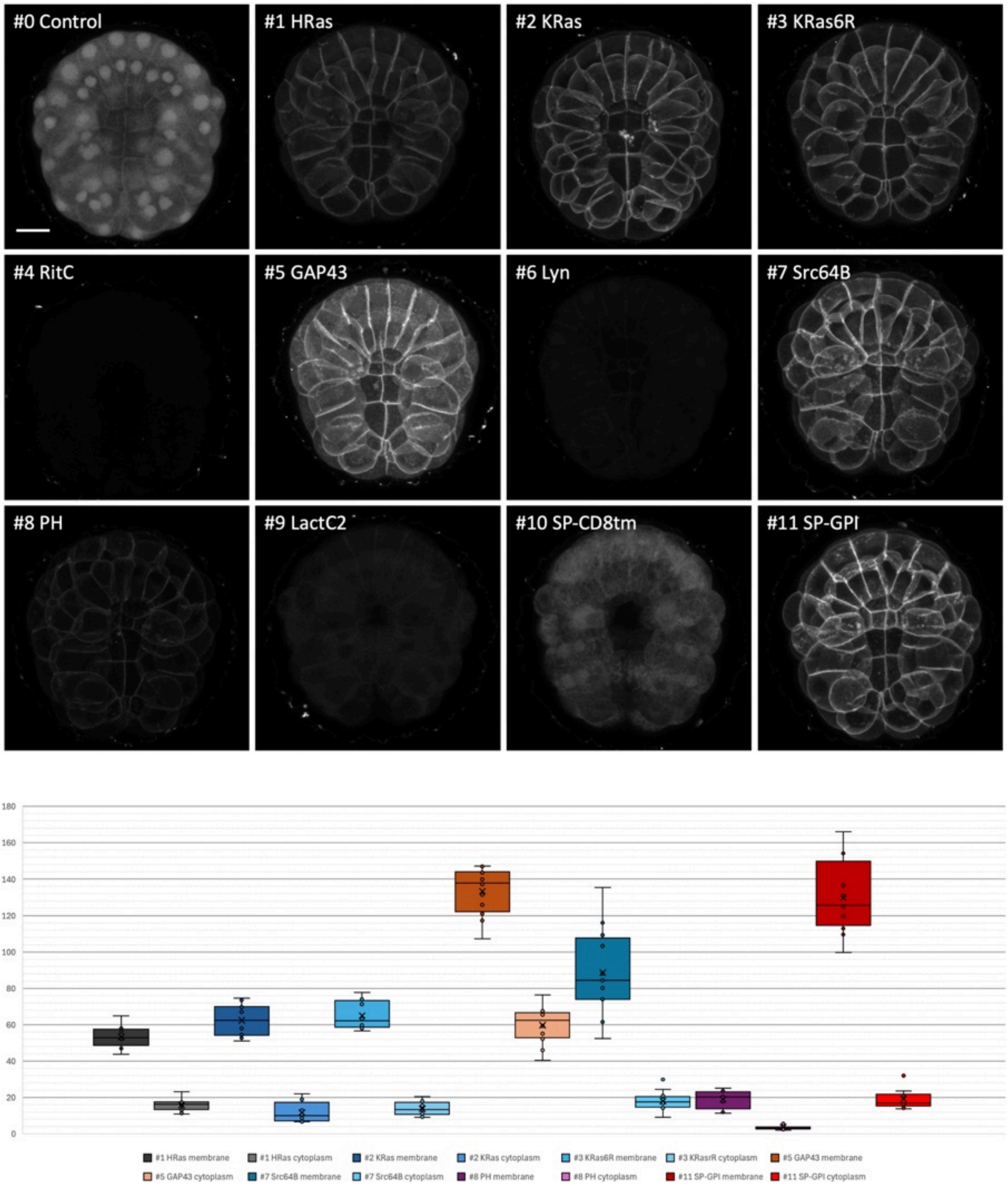

bioRxiv preprint doi: https://doi.org/10.1101/2024.11.12.623055; this version posted October 22, 2025. The copyright holder for this preprint (which was not certified by peer review) is the author/funder, who has granted bioRxiv a license to display the preprint in perpetuity. It is made available under aCC-BY 4.0 International license.

Figure S2. Quantification of cell membrane localisation in the sea urchin *Paracentrotus lividus*

(Top) mScarlet3 fluorescence in *Paracentrotus* blastula stage embryos. Images are single confocal planes on the embryo's surface; unlike the images shown in Figure 3, these images were captured and displayed with the same settings to reveal differences in fluorescence intensity. Note that #7 shows no fluorescence at the blastula stage, but gives strong membrane-localised fluorescence at the gastrula stage (see Figure 3). Scale bar, 50 µm.

(Bottom) Quantification of fluorescence at the plasma membrane and in the cell interior, as described in the Methods.

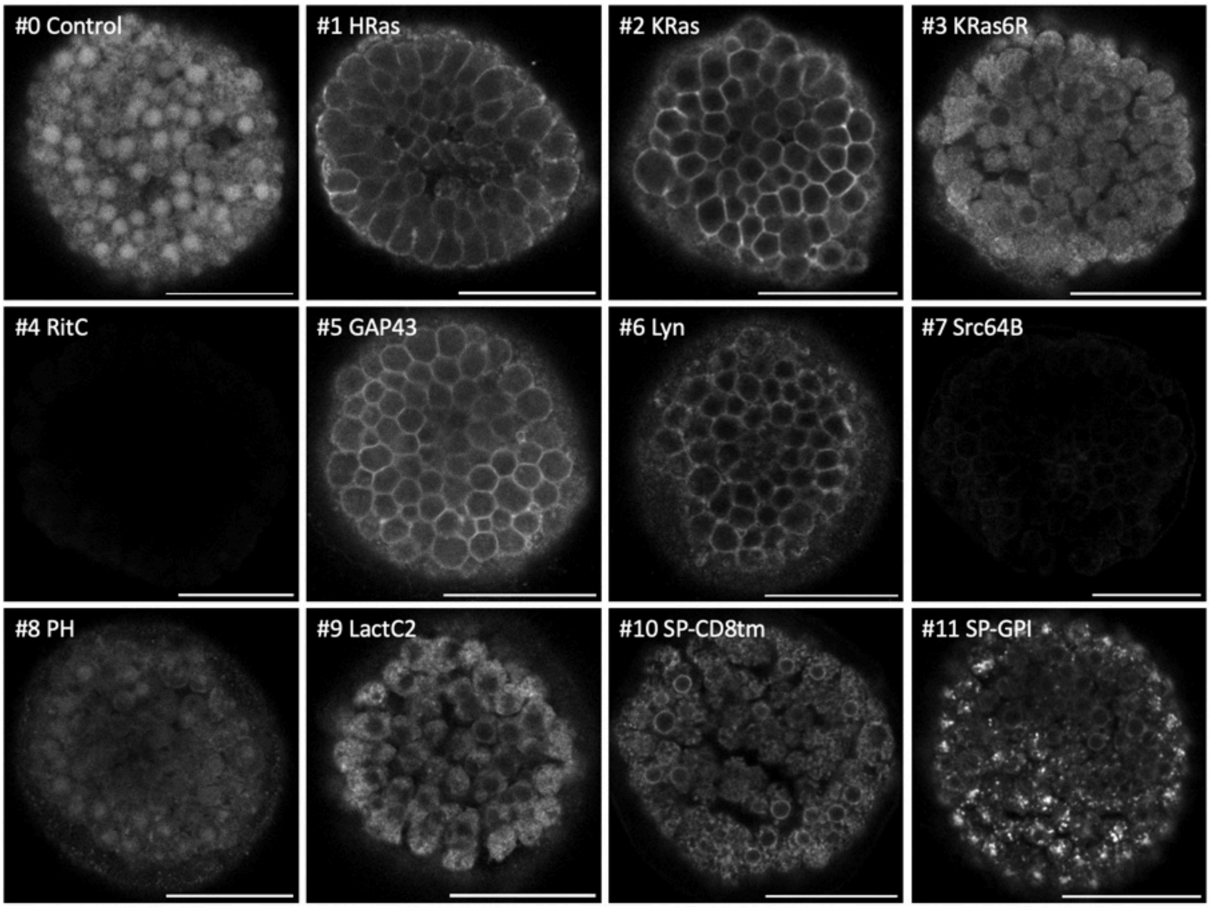

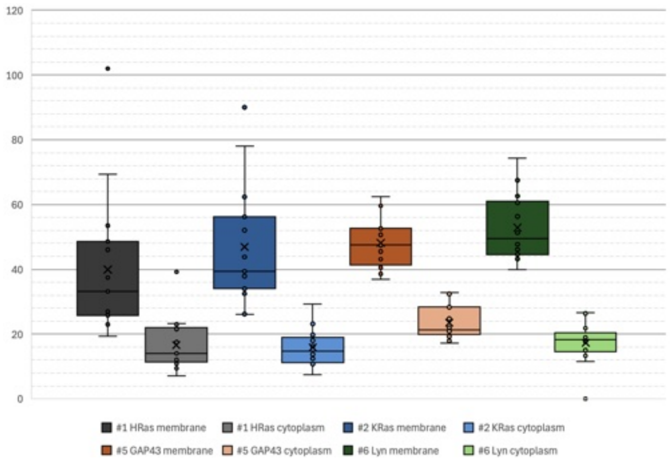

bioRxiv preprint doi: https://doi.org/10.1101/2024.11.12.623055; this version posted October 22, 2025. The copyright holder for this preprint (which was not certified by peer review) is the author/funder, who has granted bioRxiv a license to display the preprint in perpetuity. It is made available under aCC-BY 4.0 International license.

Figure S3. Localisation of membrane-tagged reporters in the beetle *Tribolium castaneum*

(A) mScarlet3 fluorescence in late blastoderm embryos of *Tribolium castaneum* injected with mRNAs of the membrane-tagged and control constructs. Some of the embryos show mosaic expression. Maximum intensity projections acquired with similar settings (see Methods). Scale bar, 50 µm.

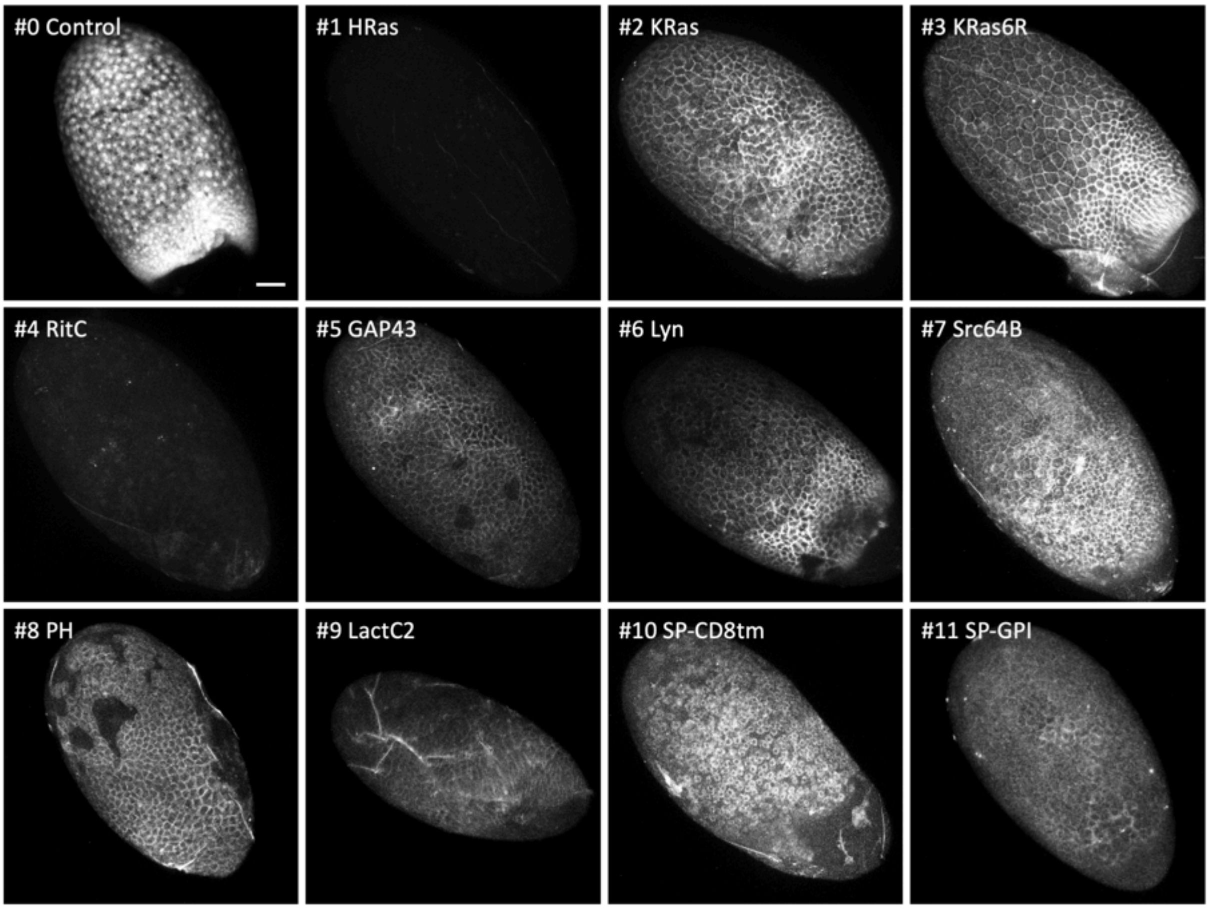

bioRxiv preprint doi: https://doi.org/10.1101/2024.11.12.623055; this version posted October 22, 2025. The copyright holder for this preprint (which was not certified by peer review) is the author/funder, who has granted bioRxiv a license to display the preprint in perpetuity. It is made available under aCC-BY 4.0 International license.

Figure S3. Localisation of membrane-tagged reporters in the beetle *Tribolium castaneum* (continued)

(B) Selected reporters imaged at higher magnification. Scale bar, 20 µm.

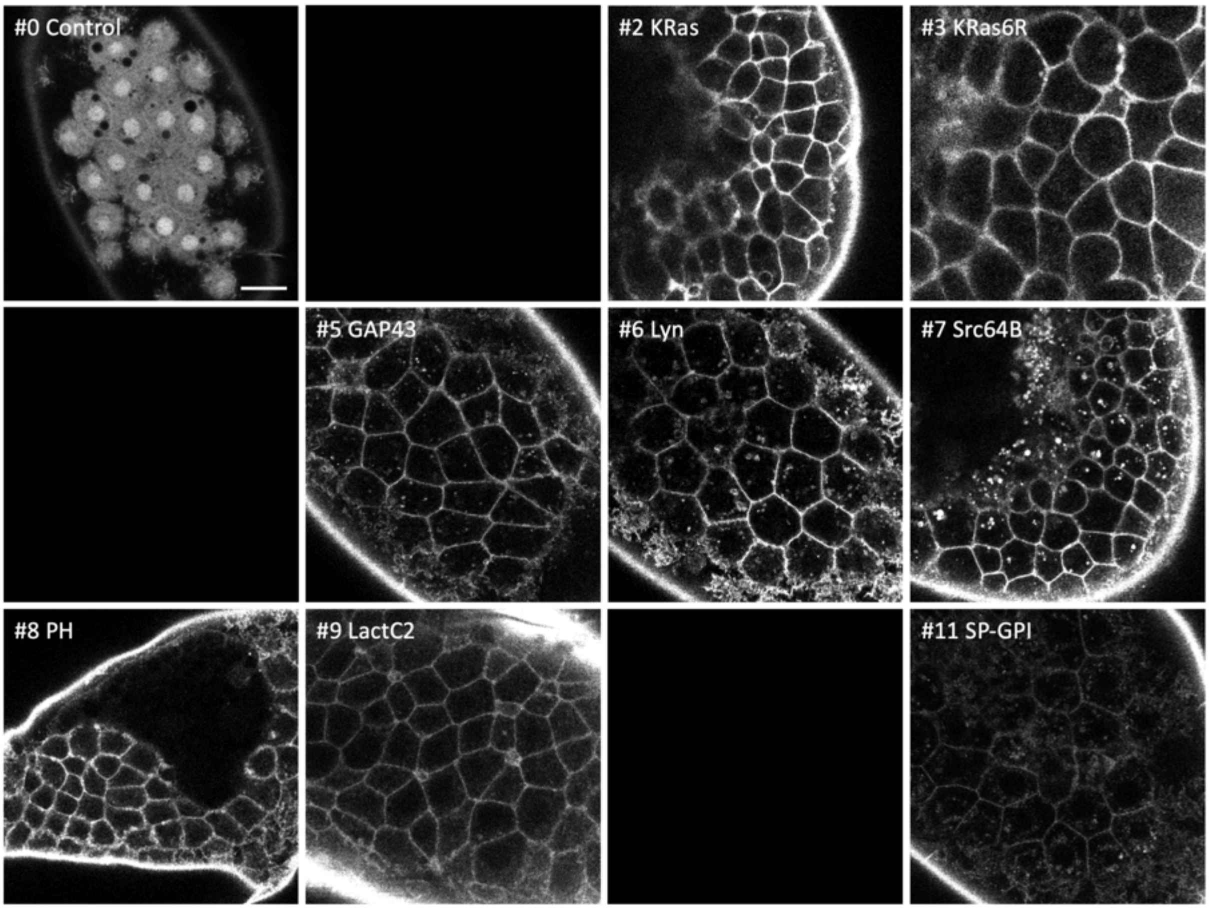

bioRxiv preprint doi: https://doi.org/10.1101/2024.11.12.623055; this version posted October 22, 2025. The copyright holder for this preprint (which was not certified by peer review) is the author/funder, who has granted bioRxiv a license to display the preprint in perpetuity. It is made available under aCC-BY 4.0 International license.

Figure S4. Quantification of cell membrane localisation in the crustacean *Parhyale hawaiensis*

(Top) mScarlet3 fluorescence in 1-day old *Parhyale* embryos, injected with mRNA of the membrane-tagged and control constructs. Only parts of each embryo express the reporter, due to uneven distribution of the injected mRNA. Unlike Figure 4, the images show maximum projections of images acquired by confocal microscopy, captured and displayed with the same settings to reveal differences in fluorescence intensity (except #0 and #10, which were captured in an independent experiment, and #4, which was captured by conventional fluorescence microscopy). Scale bars, 50 µm.

(Bottom) Quantification of fluorescence at the plasma membrane and in the cell interior, as described in the Methods.

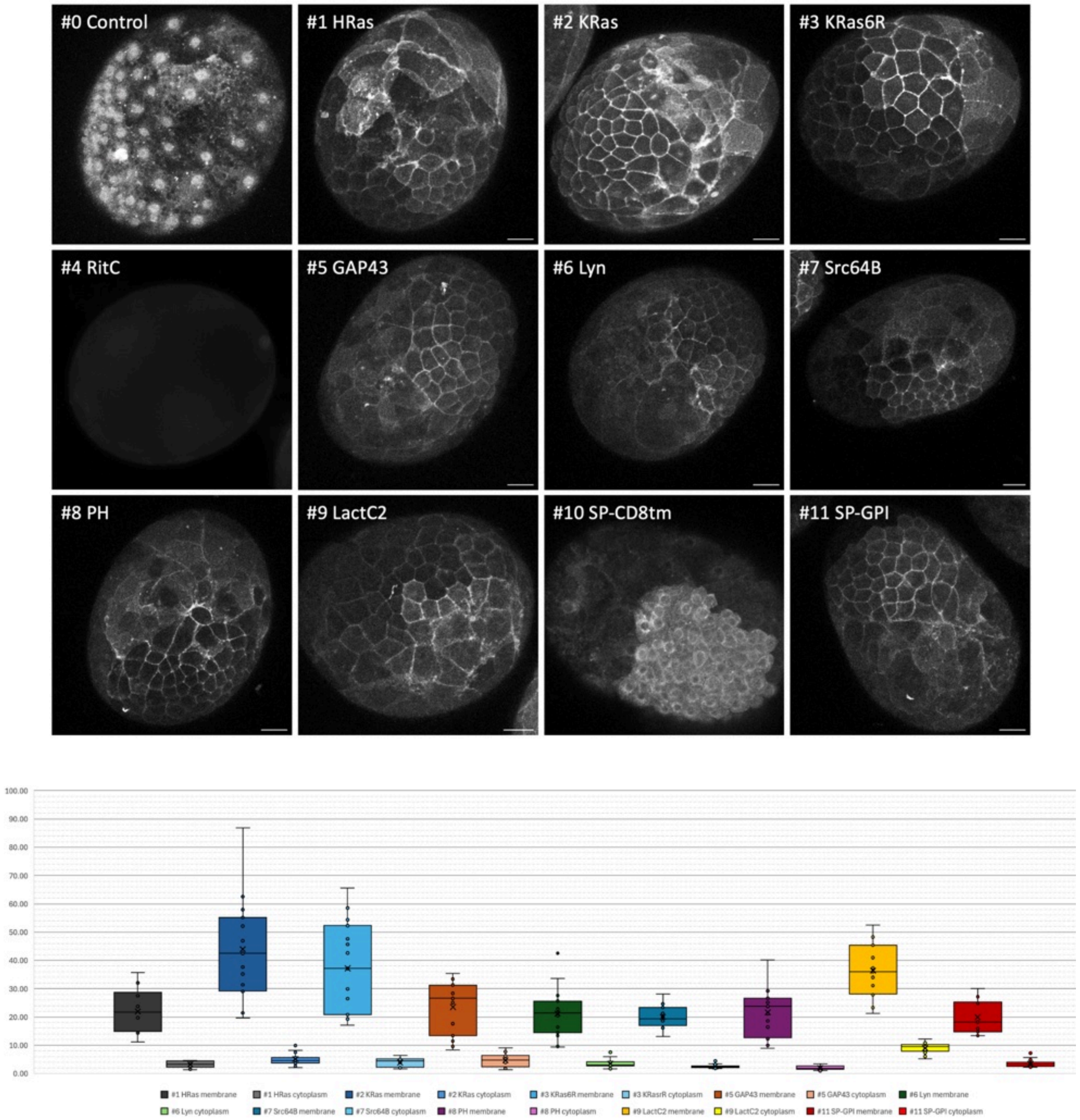

bioRxiv preprint doi: https://doi.org/10.1101/2024.11.12.623055; this version posted October 22, 2025. The copyright holder for this preprint (which was not certified by peer review) is the author/funder, who has granted bioRxiv a license to display the preprint in perpetuity. It is made available under aCC-BY 4.0 International license.

Figure S5. Localisation of membrane-tagged reporters in the nematode *Caenorhabditis elegans*

mScarlet3 fluorescence in early embryos of *Caenorhabditis elegans* after injecting the mRNAs of the membrane-tagged and control constructs in the syncytial gonads of their parents. The images were acquired using the same settings, but brightness and contrast were adjusted to reveal weak fluorescence. Scale bar, 20 µm.

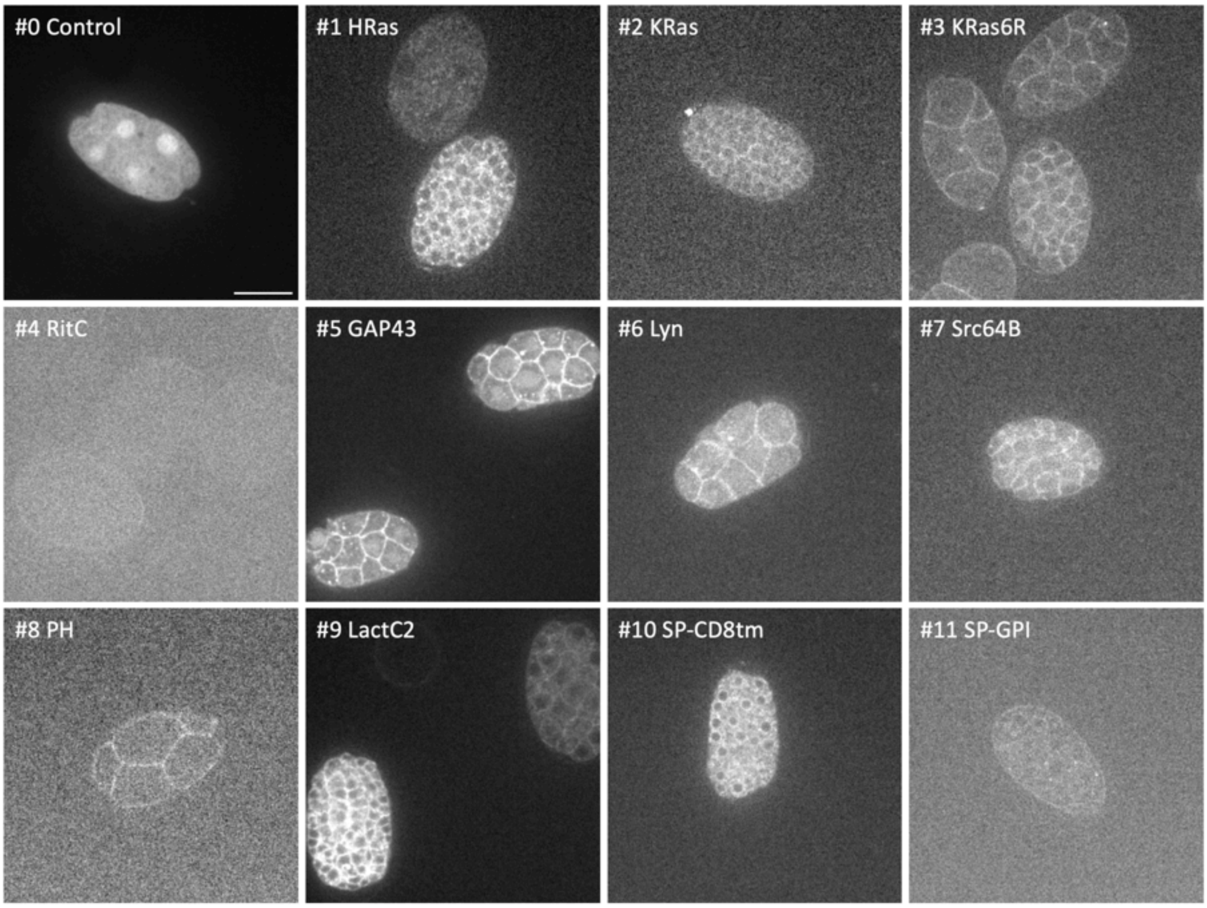

bioRxiv preprint doi: https://doi.org/10.1101/2024.11.12.623055; this version posted October 22, 2025. The copyright holder for this preprint (which was not certified by peer review) is the author/funder, who has granted bioRxiv a license to display the preprint in perpetuity. It is made available under aCC-BY 4.0 International license.

Figure S6. Localisation of membrane-tagged reporters in annelid *Platynereis dumerilii* embryos

mScarlet3 fluorescence in *Platynereis* embryos injected with mRNA at the 1- or 2-cell stage. Images show maximum intensity projections from light sheet microscopy, captured with different laser intensities (see Methods), at various embryonic stages: #9 at 6 hpf; #0, 1, 3, 10, 11 at 9 hpf; #7 at 10 hpf; #2, 4, 6 at 11.5 hpf; #5 at 14.5 hpf, #8 at 23 hpf (hpf, hours post fertilisation). Some embryos are mosaic because injections were performed at the 2-cell stage. Scale bar, 50 µm.

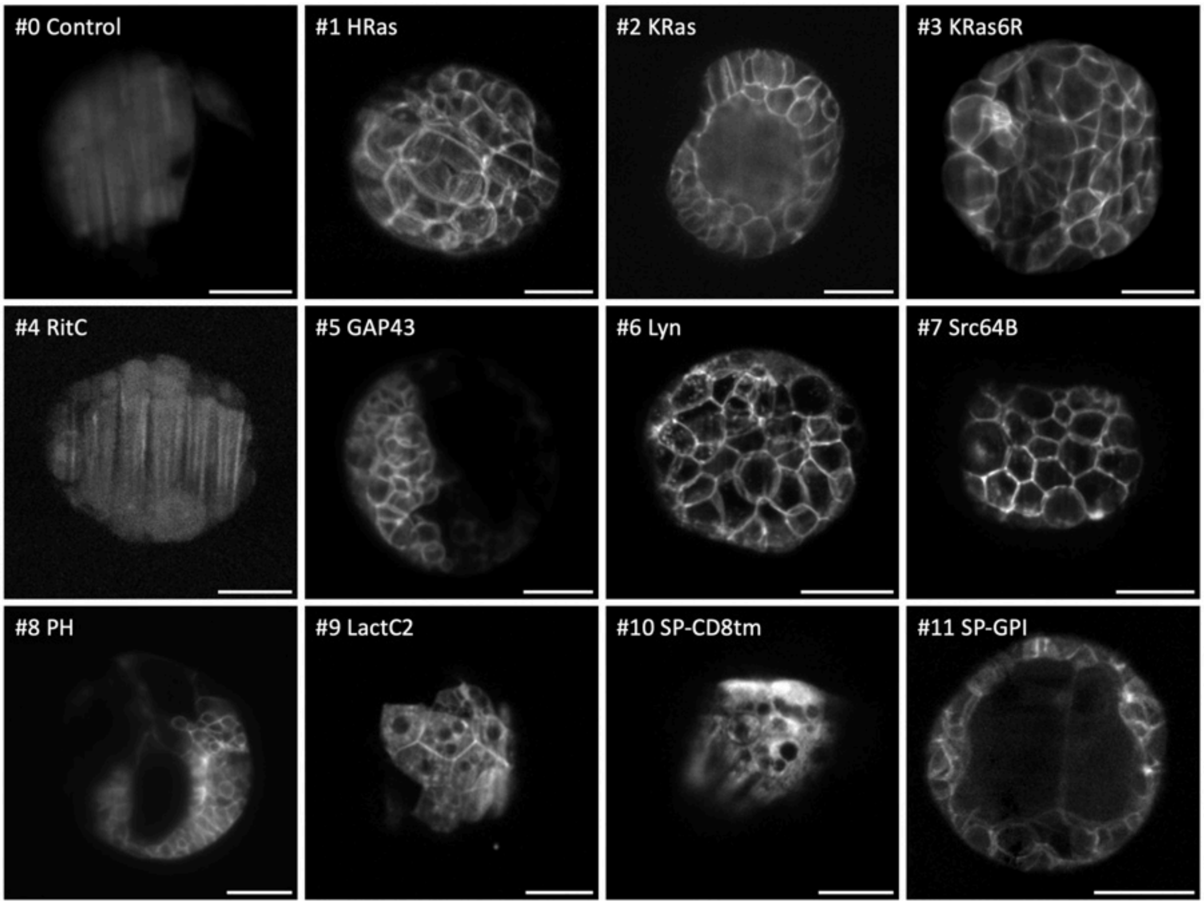

bioRxiv preprint doi: https://doi.org/10.1101/2024.11.12.623055; this version posted October 22, 2025. The copyright holder for this preprint (which was not certified by peer review) is the author/funder, who has granted bioRxiv a license to display the preprint in perpetuity. It is made available under aCC-BY 4.0 International license.

Figure S7. Localisation of membrane-tagged reporters in annelid *Platynereis dumerilii* larvae

mScarlet3 fluorescence in *Platynereis* larvae injected with mRNA of the membrane-tagged reporters at the 1 cell stage, compared with an uninjected larva (wt). Images show maximum intensity projections of the ventral half of each larva imaged by light sheet microscopy (see Methods), between 2.5 and 7 days post fertilisation (#6, #9 and #11 at 2.5 dpf; #3 and #4 at 3dpf; wt, #0, #2, #7, #8 and #10 at 4 dpf; #5 at 6 dpf; #1 at 7 dpf). Autofluorescence can be seen in the parapodial glands (as shown by an arrow in wt). Reporters #1 HRas, #2 KRas, #3 KRas6R, #5 GAP43, #6 Lyn, #7 Scr64B, #8 PH and #9 LactC2 showed fluorescence in the brain and ventral nerve cord (marked by arrowheads in #6). Membrane labelling outside of the nervous system was particularly visible in larvae injected with reporters #6 and #9 (higher magnification images shown in the lower left corner). Reporters #0, #4, #10 and #11 did not show a clear fluorescent signal. With some reporters (particularly #11), injecting large amounts of mRNA delayed development. The anterior of larvae is up, ventral views. Scale bars, 50 µm.

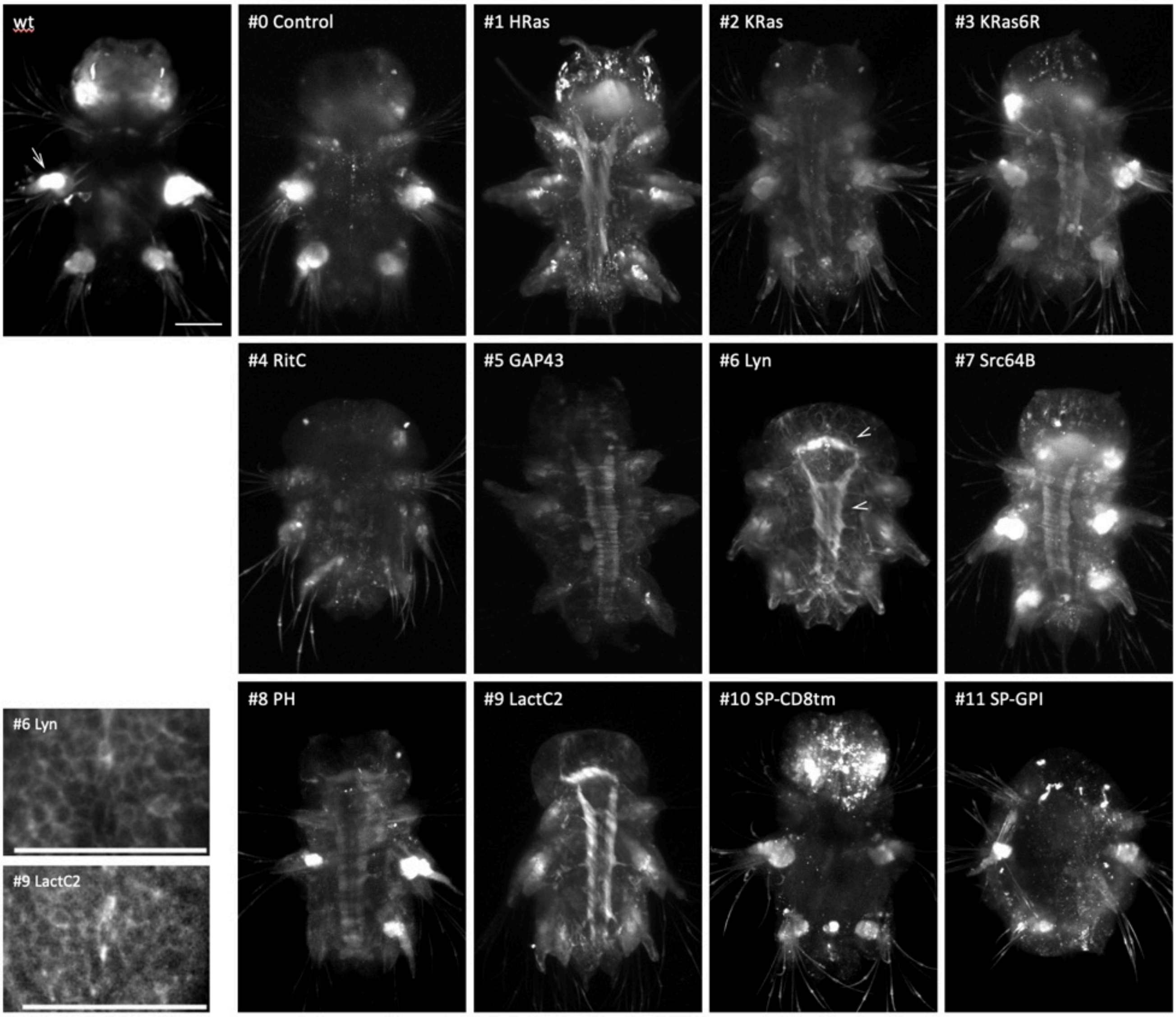

bioRxiv preprint doi: https://doi.org/10.1101/2024.11.12.623055; this version posted October 22, 2025. The copyright holder for this preprint (which was not certified by peer review) is the author/funder, who has granted bioRxiv a license to display the preprint in perpetuity. It is made available under aCC-BY 4.0 International license.

Figure S8. Localisation of membrane-tagged reporters in the flatworm *Macrostomum lignano*

mScarlet3 fluorescence in *Macrostomum* embryos injected with mRNA at the 1-cell stage and imaged 16 hours post injection. Images show confocal optical sections through the embryos, acquired using the same settings. Scale bar, 25 µm.

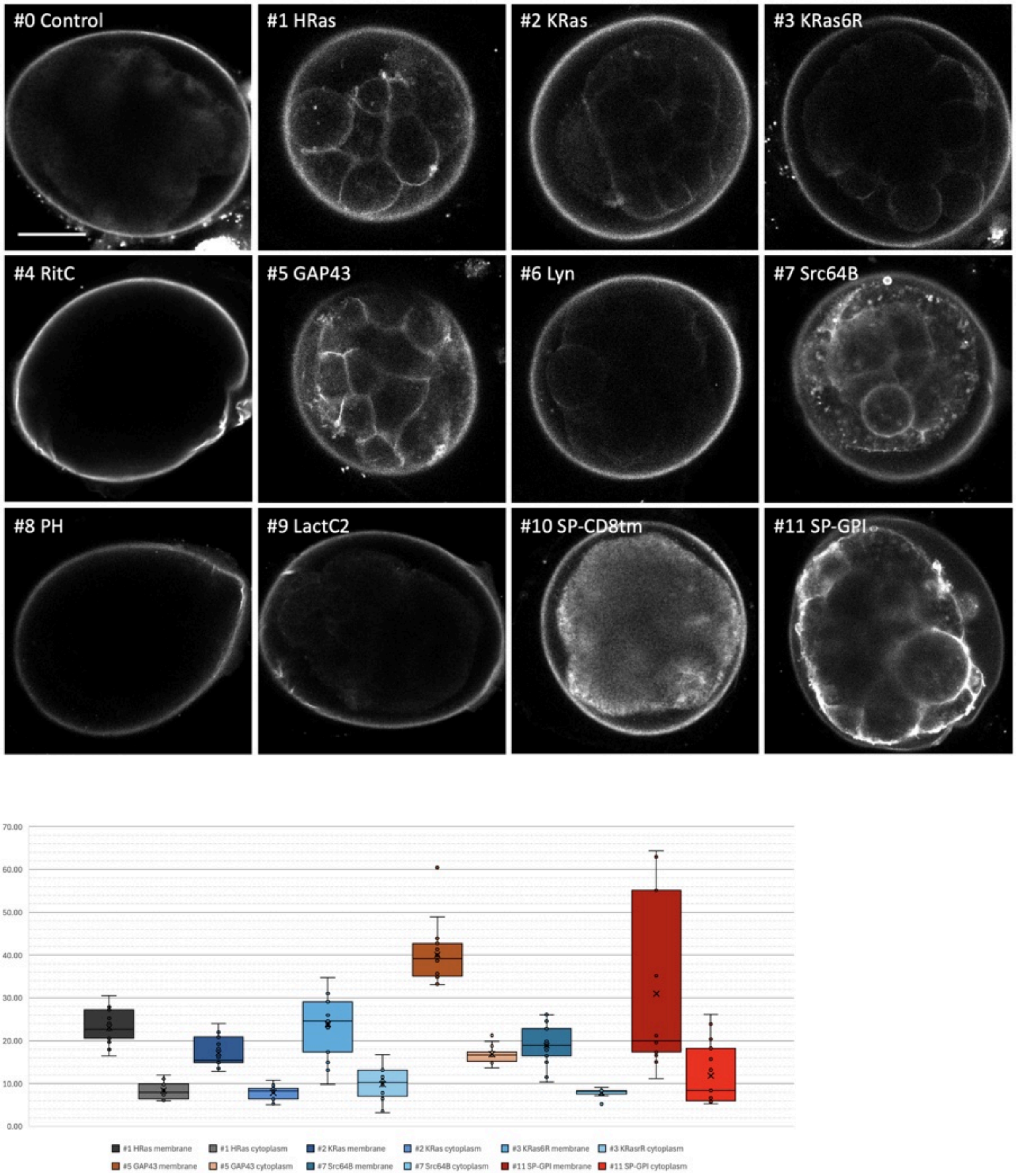

bioRxiv preprint doi: https://doi.org/10.1101/2024.11.12.623055; this version posted October 22, 2025. The copyright holder for this preprint (which was not certified by peer review) is the author/funder, who has granted bioRxiv a license to display the preprint in perpetuity. It is made available under aCC-BY 4.0 International license.

Figure S9. Quantification of cell membrane localisation in hydrozoan *Clytia hemisphaerica* embryos

(Top) Same as in Figure 5, but images were captured and displayed with the same settings, to reveal differences in fluorescence intensity. Scale bar, 50 µm.

(Bottom) Quantification of fluorescence at the plasma membrane and in the cell interior, as described in the Methods.

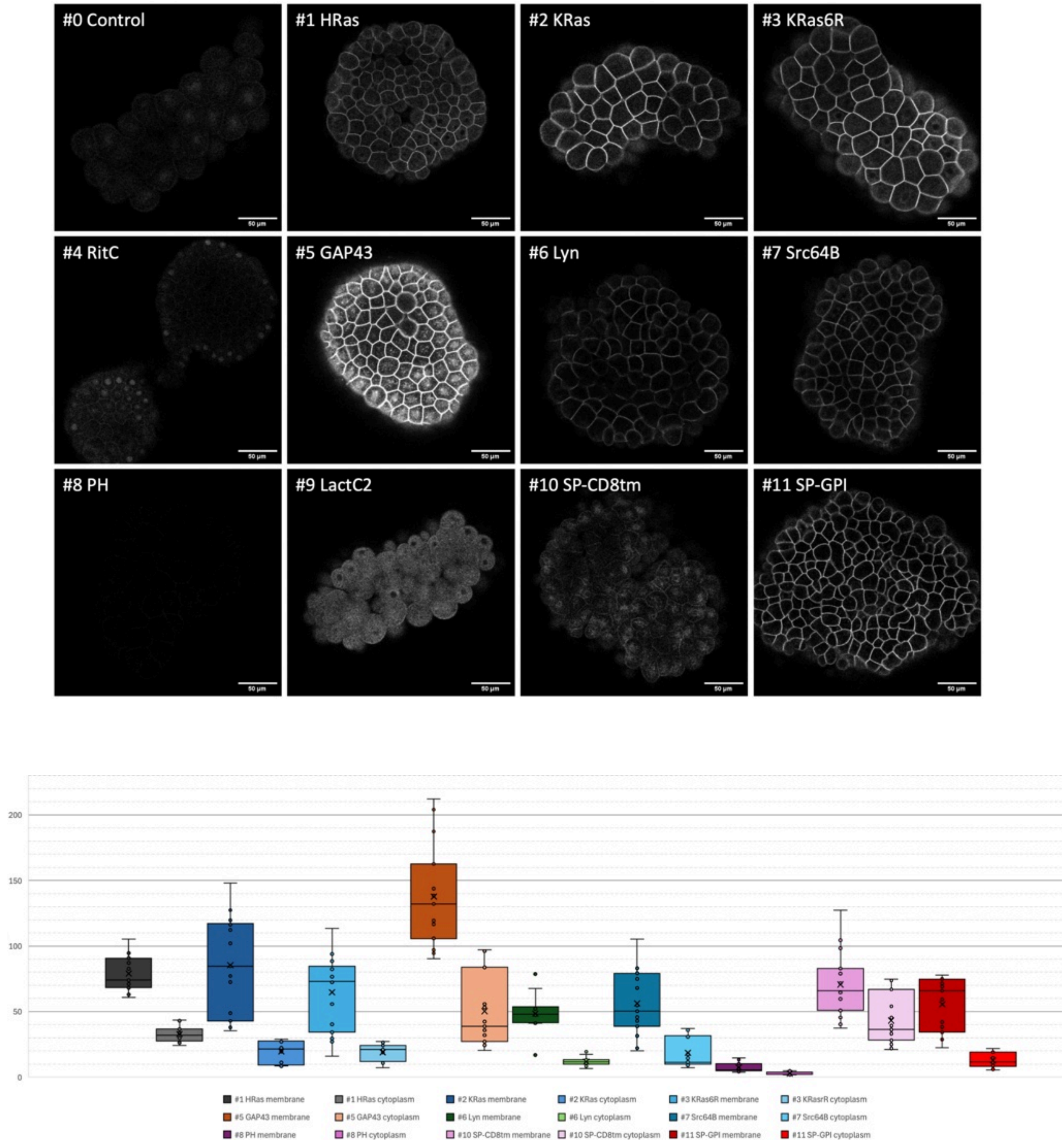

bioRxiv preprint doi: https://doi.org/10.1101/2024.11.12.623055; this version posted October 22, 2025. The copyright holder for this preprint (which was not certified by peer review) is the author/funder, who has granted bioRxiv a license to display the preprint in perpetuity. It is made available under aCC-BY 4.0 International license.

Figure S10. Localisation of membrane-tagged reporters in hydrozoan *Clytia hemisphaerica* polyps

mScarlet3 fluorescence in *Clytia* primary polyps a few hours after settlement, 3 days after mRNA injection into oocytes. The images are from a single confocal plane capturing the outer epidermal layer of the polyp, on the oral side. They were acquired with the same settings, so fluorescent intensities are comparable across panels. Scale bar, 10 µm.

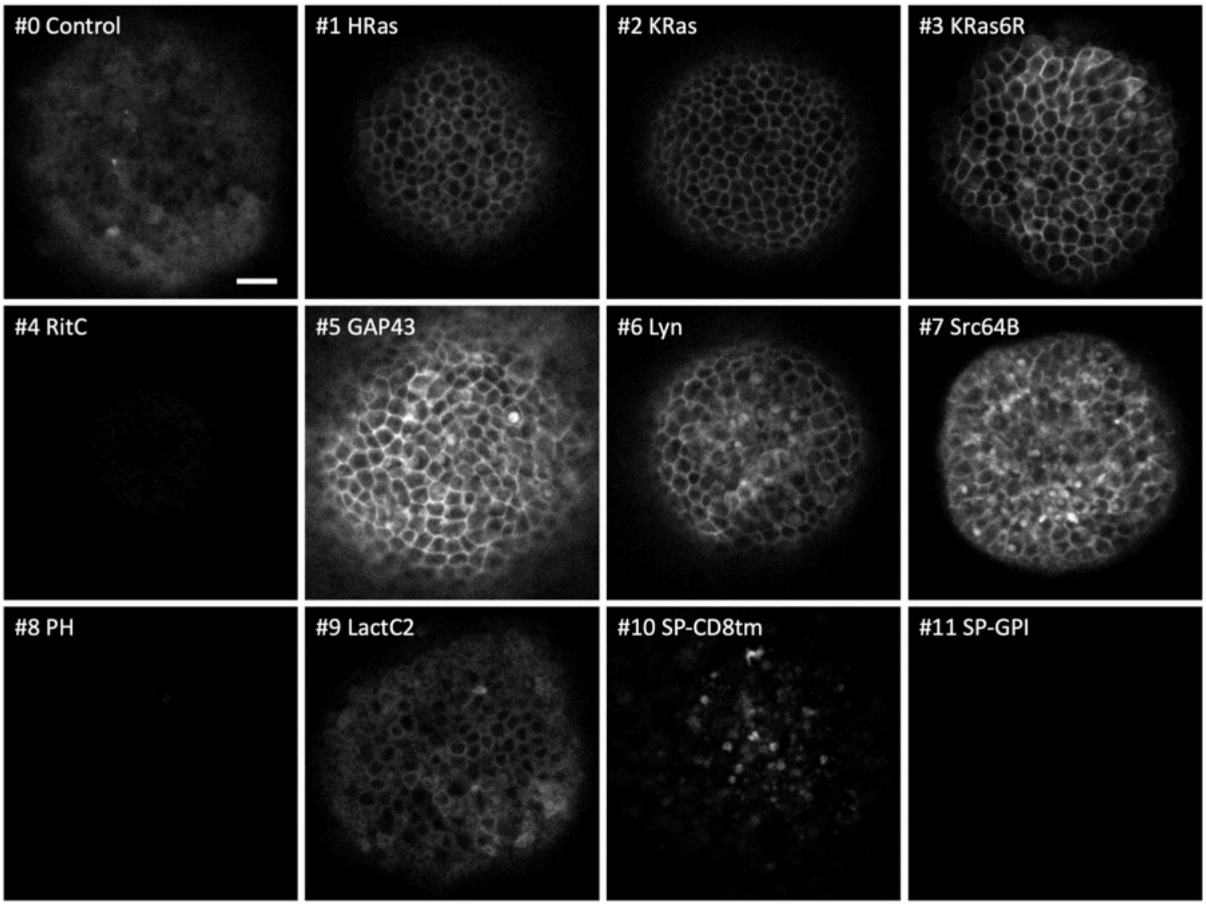

bioRxiv preprint doi: https://doi.org/10.1101/2024.11.12.623055; this version posted October 22, 2025. The copyright holder for this preprint (which was not certified by peer review) is the author/funder, who has granted bioRxiv a license to display the preprint in perpetuity. It is made available under aCC-BY 4.0 International license.

Figure S11. Localisation of membrane-tagged reporters in scyphozoan *Pelagia noctiluca* embryos

mScarlet fluorescence in *Pelagia noctiluca* gastrula stage embryos, 22 to 26 h after mRNA injection in zygotes. The images show a single confocal plane capturing the outer epidermal layer of the gastrula. They were captured with the same settings, so fluorescent intensities are comparable across panels. Scale bar, 20 µm.

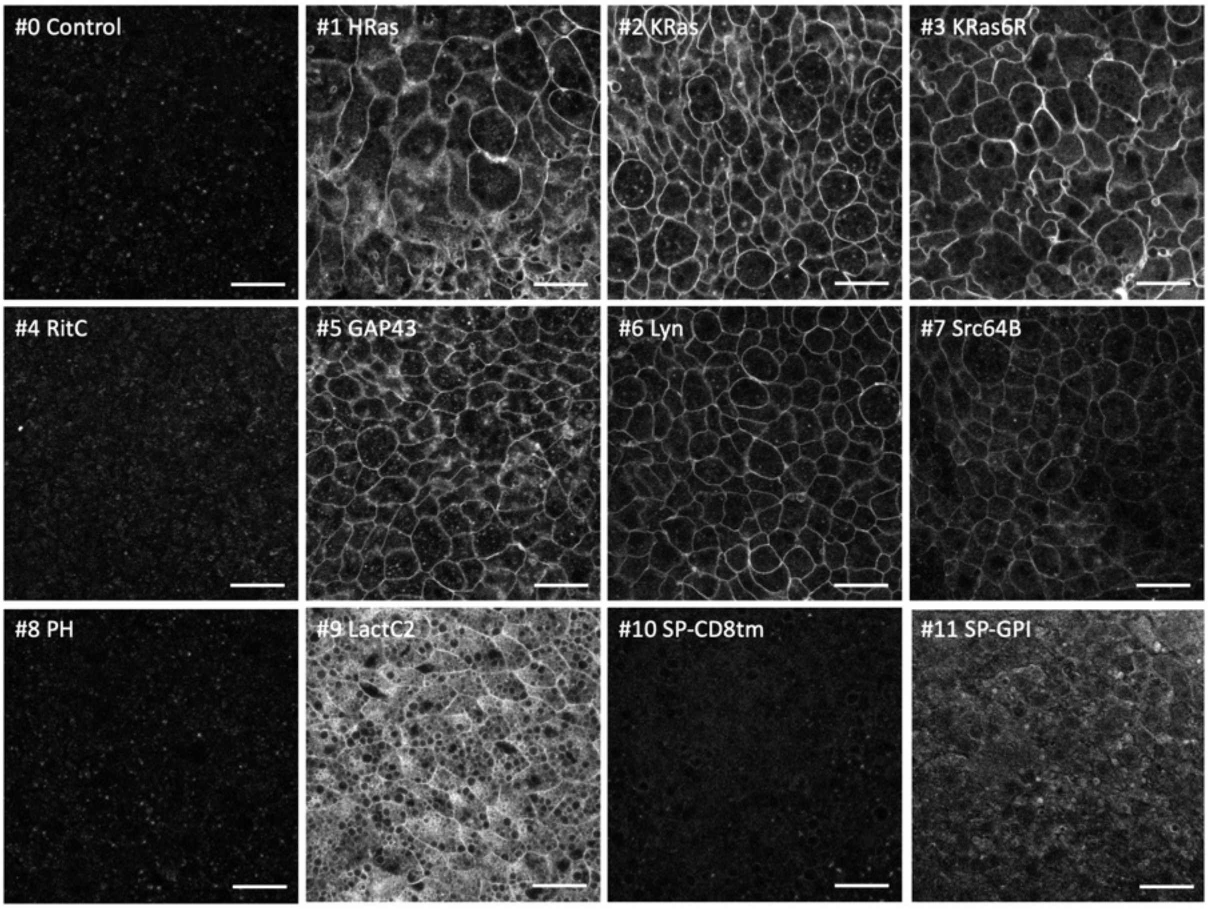

bioRxiv preprint doi: https://doi.org/10.1101/2024.11.12.623055; this version posted October 22, 2025. The copyright holder for this preprint (which was not certified by peer review) is the author/funder, who has granted bioRxiv a license to display the preprint in perpetuity. It is made available under aCC-BY 4.0 International license.

Figure S12. Localisation of membrane-tagged reporters in scyphozoan *Pelagia noctiluca* planulae

mScarlet3 fluorescence in *Pelagia noctiluca* planula larvae, 46 to 50 h after mRNA injection in zygotes. The images show a single confocal plane capturing the outer epidermal layer of the planula. They were captured with the same settings, so fluorescent intensities are comparable across panels. Scale bar, 20µm.

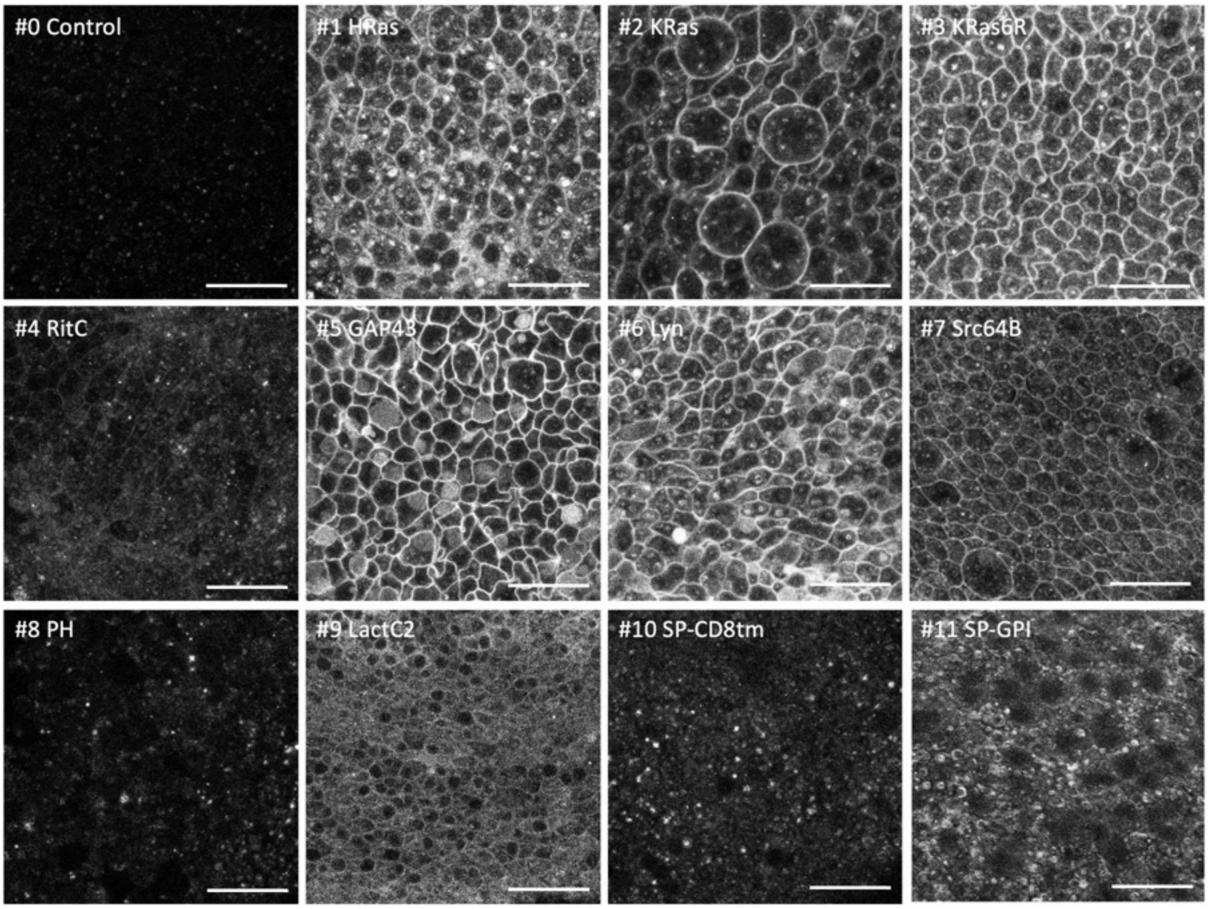

bioRxiv preprint doi: https://doi.org/10.1101/2024.11.12.623055; this version posted October 22, 2025. The copyright holder for this preprint (which was not certified by peer review) is the author/funder, who has granted bioRxiv a license to display the preprint in perpetuity. It is made available under aCC-BY 4.0 International license.

Figure S13. Localisation of membrane-tagged reporters in the anthozoan *Nematostella vectensis*

mScarlet3 localisation in *Nematostella* late gastrula-stage embryos, approximately 30 hours after mRNA injection. The embryos were fixed and stained with antibodies for mScarlet3 (shown in magenta) and with phalloidin (labelling actin, in green), as described in the Methods. The images show a single confocal plane across the surface of the embryo. They were acquired with the same settings, except that gain was increased for mRNAs giving weaker fluorescence (as indicated on each image). The best membrane-localising construct was #1, which gave the strongest staining of the plasma membrane, including the microvilli and cilia. Scale bars, 20 µm.

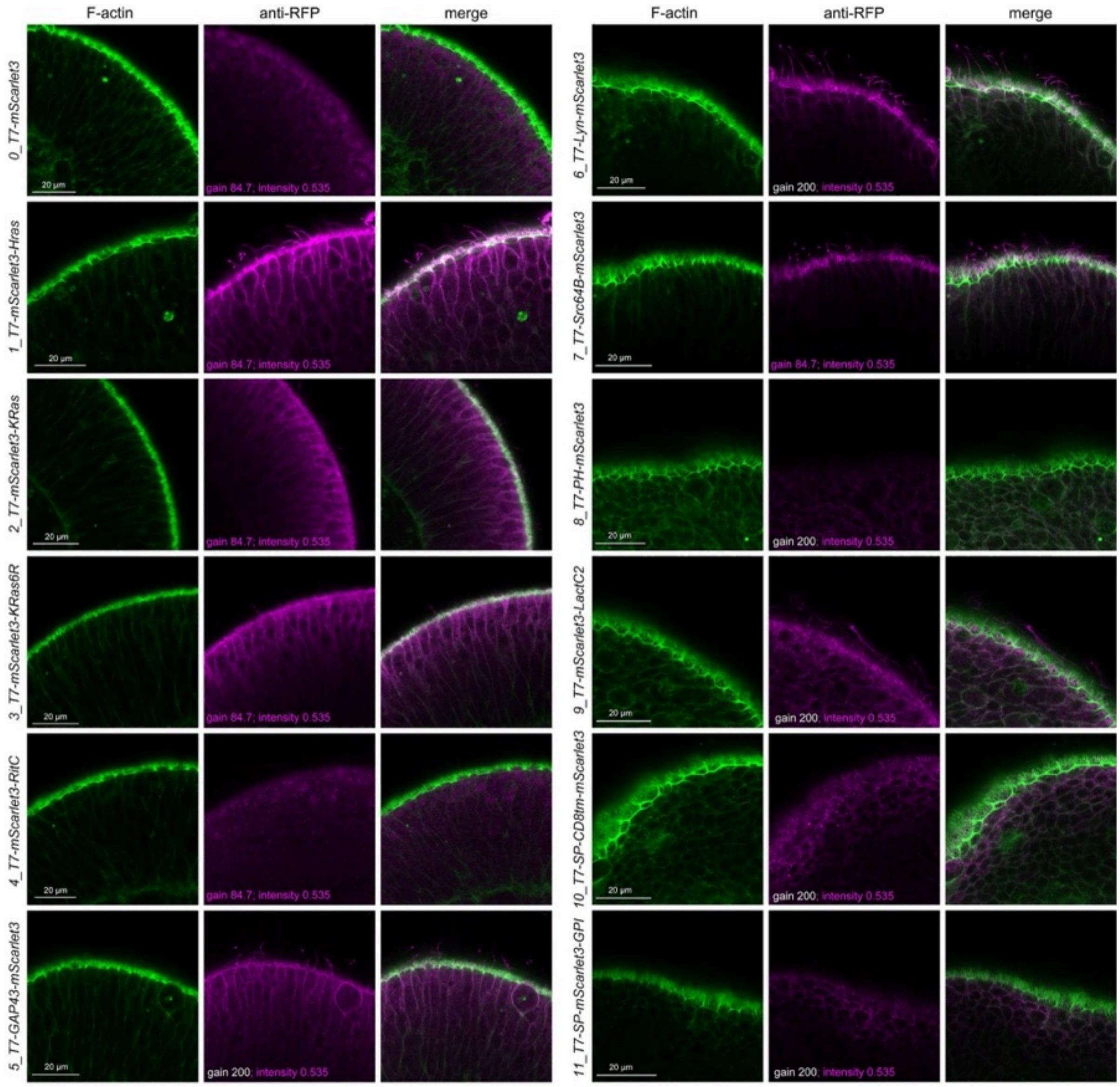

bioRxiv preprint doi: https://doi.org/10.1101/2024.11.12.623055; this version posted October 22, 2025. The copyright holder for this preprint (which was not certified by peer review) is the author/funder, who has granted bioRxiv a license to display the preprint in perpetuity. It is made available under aCC-BY 4.0 International license.

Figure S14. Localisation of tagged proteins in abnormal *Paracentrotus* embryos

Three reporters that did not give plasma membrane localisation of mScarlet3 in normal blastula stage embryos (#3, #8 and #9) and one that was only detected at later stages (#7), showed localisation at the plasma membrane in a batch of abnormal *Paracentrotus* blastulae, probably arising from polyspermy (see Methods). Images show single confocal planes. Normal blastulae are shown on the left, abnormal blastulae on the right. Images show single confocal planes and have been adjusted in brightness and contrast. Scale bars, 50 µm.

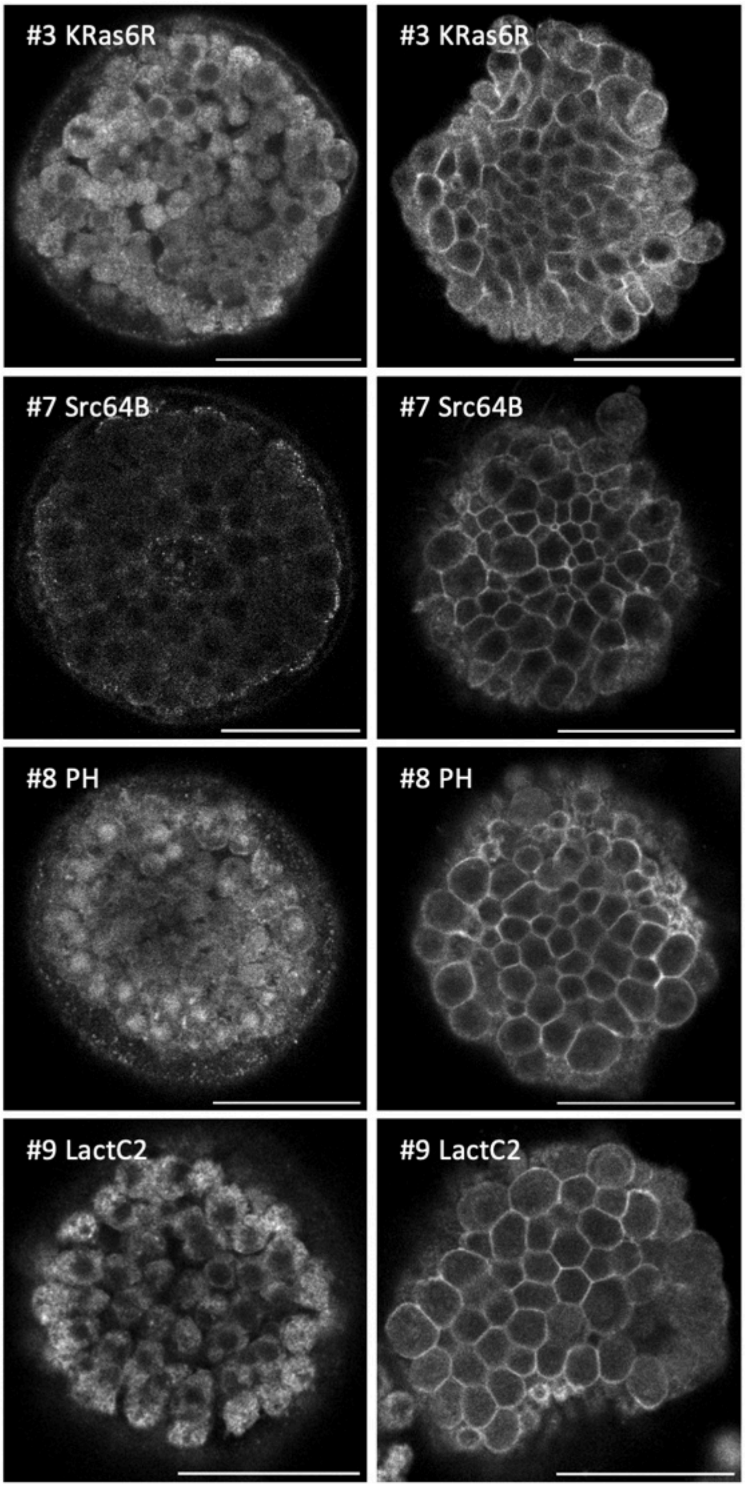

bioRxiv preprint doi: https://doi.org/10.1101/2024.11.12.623055; this version posted October 22, 2025. The copyright holder for this preprint (which was not certified by peer review) is the author/funder, who has granted bioRxiv a license to display the preprint in perpetuity. It is made available under aCC-BY 4.0 International license.

Figure S15. Improved reporters using endogenous UTRs and codon optimisation in *Clytia*

Using codon-optimised mCherry (adapted to the codon usage of *Clytia*, Weissbourd et al. 2021) flanked by 5' and 3' UTRs of a *Clytia hemispherica* genes (Uveira et al. 2024) can increase the fluorescence intensity of KRas and PH tagged reporters.

(Top) *Clytia hemisphaerica* primary polyps injected with mRNA of the #2 KRas reporter (left) or codon-optimised mCherry, tagged with the same KRas tag and flanked by *Clytia* UTRs (CC-KRas, right). The CC-KRas reporter gave at least 5-fold brighter fluorescence than #2 KRas (the image was acquired with 5-fold lower exposure). Scale bars, 5 µm.

(Bottom) *Clytia hemisphaerica* blastula-stage embryos injected with mRNA of the #8 PH reporter (left) or codon-optimised mCherry, fused with a codon-optimised PH tag (Uveira et al. 2024) and flanked by *Clytia* UTRs (PH-CC, right). The PH-CC reporter gave at least 3-fold brighter fluorescence than #8 PH (the image was acquired with 3-fold lower exposure). Scale bars, 50 µm.

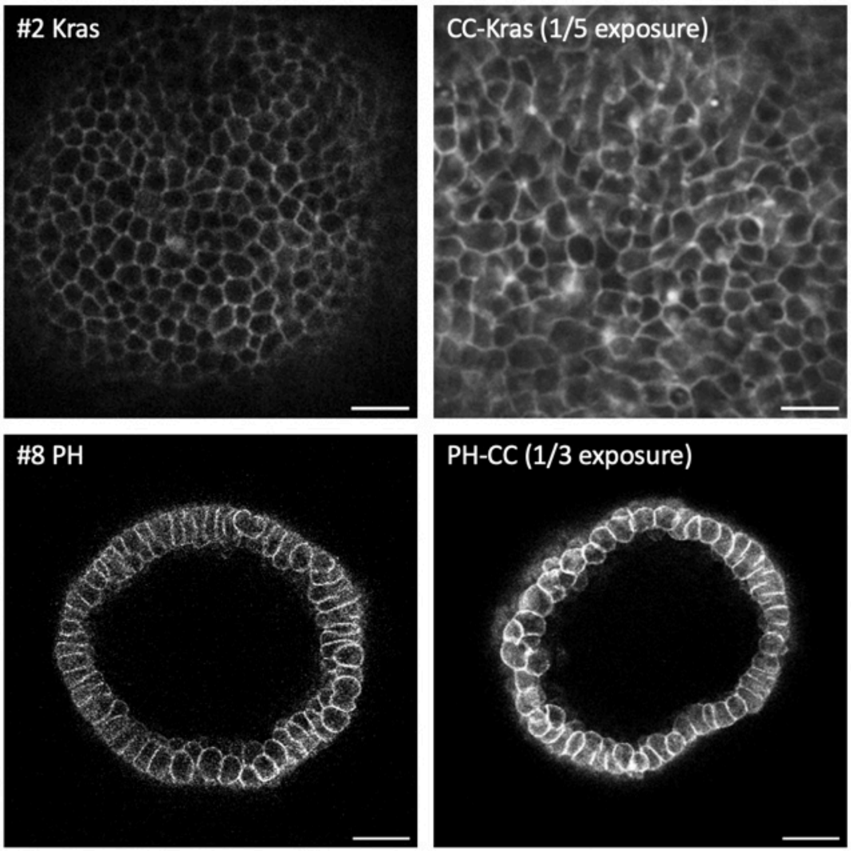

bioRxiv preprint doi: https://doi.org/10.1101/2024.11.12.623055; this version posted October 22, 2025. The copyright holder for this preprint (which was not certified by peer review) is the author/funder, who has granted bioRxiv a license to display the preprint in perpetuity. It is made available under aCC-BY 4.0 International license.

Video S1. Dynamics of SP-CD8tm-mScarlet3 localisation (#10) in *Parhyale* embryos

Parhyale embryo injected with #10 SP-CD8tm mRNA at the 1-cell stage and imaged on a confocal microscope at 20-minute time intervals. The video shows a maximum projection of multiple confocal sections, starting approximately 24 hours after injection. SP-CD8tm-mScarlet3 is partly localised on the plasma membrane at the start of the video, but it gradually shifts to a perinuclear localisation. During mitosis, SP-CD8tm-mScarlet3 can be seen aggregating in discrete regions within the cell. Different levels of fluorescence across the embryo likely reflect different amounts of mRNA inherited by each early blastomere. Scale bar, 20 µm.

The video is available at this link: https://doi.org/10.5281/zenodo.17401844
